# Direct m6A recognition by IMP1 underlays an alternative model of target selection for non-canonical methyl-readers

**DOI:** 10.1093/nar/gkad534

**Published:** 2023-06-28

**Authors:** Giuseppe Nicastro, Giancarlo Abis, Pierre Klein, Sofia Esteban-Serna, Christopher Gallagher, Belen Chaves-Arquero, Yuyang Cai, Angelo Miguel Figueiredo, Stephen R Martin, Rickie Patani, Ian A Taylor, Andres Ramos

**Affiliations:** Macromolecular Structure Laboratory, The Francis Crick Institute, 1 Midland Road, London NW1 1AT, UK; Division of Biosciences, Institute of Structural and Molecular Biology, University College London, London, UK; Division of Biosciences, Institute of Structural and Molecular Biology, University College London, London, UK; Division of Biosciences, Institute of Structural and Molecular Biology, University College London, London, UK; Division of Biosciences, Institute of Structural and Molecular Biology, University College London, London, UK; Division of Biosciences, Institute of Structural and Molecular Biology, University College London, London, UK; Division of Biosciences, Institute of Structural and Molecular Biology, University College London, London, UK; Division of Biosciences, Institute of Structural and Molecular Biology, University College London, London, UK; Structural Biology Technology Platform, The Francis Crick Institute, 1 Midland Rd, London NW1 1AT, UK; Human Stem Cells and Neurodegeneration Laboratory, The Francis Crick Institute, 1 Midland Road, London NW1 1AT, UK; Macromolecular Structure Laboratory, The Francis Crick Institute, 1 Midland Road, London NW1 1AT, UK; Division of Biosciences, Institute of Structural and Molecular Biology, University College London, London, UK

## Abstract

m6A methylation provides an essential layer of regulation in organismal development, and is aberrant in a range of cancers and neuro-pathologies. The information encoded by m6A methylation is integrated into existing RNA regulatory networks by RNA binding proteins that recognise methylated sites, the m6A readers. m6A readers include a well-characterised class of dedicated proteins, the YTH proteins, as well as a broader group of multi-functional regulators where recognition of m6A is only partially understood. Molecular insight in this recognition is essential to build a mechanistic understanding of global m6A regulation. In this study, we show that the reader IMP1 recognises the m6A using a dedicated hydrophobic platform that assembles on the methyl moiety, creating a stable high-affinity interaction. This recognition is conserved across evolution and independent from the underlying sequence context but is layered upon the strong sequence specificity of IMP1 for GGAC RNA. This leads us to propose a concept for m6A regulation where methylation plays a context-dependent role in the recognition of selected IMP1 targets that is dependent on the cellular concentration of available IMP1, differing from that observed for the YTH proteins.

## Graphical Abstract

**Graphical Abstract:**
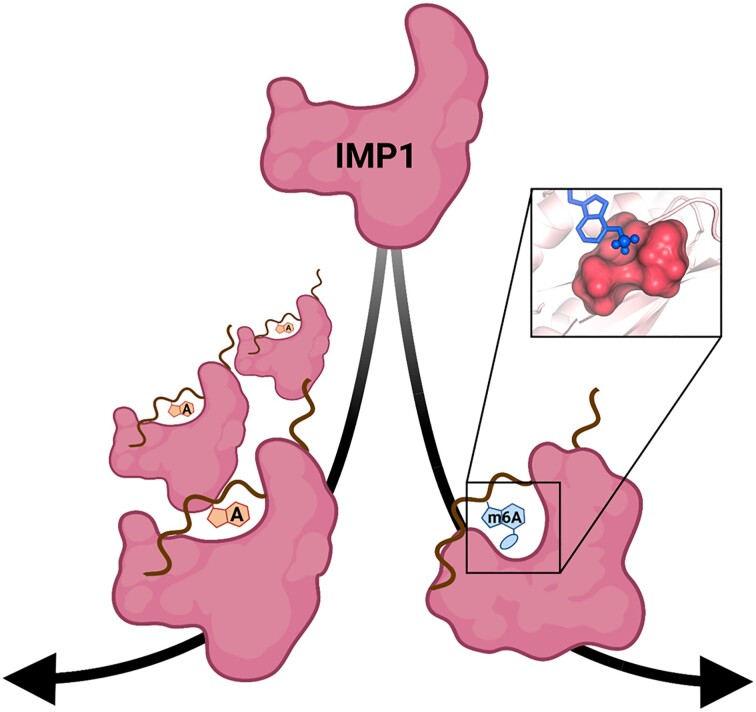

## INTRODUCTION

*N* ^6^-methyladenosine (m6A) is an internal, common mRNA modification in eukaryotes (1). m6A methylation varies according to cell type and in response to signalling and plays an essential role in organismal development. Importantly, the dysfunction of m6A regulatory pathways is related to a range of diseases, including neurological and immunological disorders, obesity and numerous cancers (1–3).

m6A sites are typically located within RAC/DRACH sequences and enriched in mRNA 3′UTRs, in particular in proximity to the stop codon (1,2,4,5). m6A methylated RNA (m6A-RNA) is bound by m6A readers, a set of RNA-binding proteins that provide a functional link with pathways regulating mRNA processing, export, localisation, stability and translation (1,6). A well-studied class of m6A readers recognises the m6A moiety through a dedicated module, the YT521-B homology (YTH) domain. This recognition increases RNA-binding affinity by 50–100-fold (6,7) and recruits such ‘canonical’ readers to the target sites.

Recent studies have shown that the scope of m6A regulation is expanded by additional ‘non-canonical’ m6A reader proteins, whose m6A-mediated activity has been linked to a number of severe pathologies (8,9). Non-canonical readers do not contain a YTH domain but instead include other RNA-binding domains, and have been reported to recognize m6A-RNA both directly and indirectly, depending on the individual proteins (1,10–13). However, how, at the molecular level, non-canonical readers recognize m6A methylation is only partially understood and remains a key question to rationalize the function and regulation of this group of proteins.

One important non-canonical reader is the insulin-like growth factor 2 mRNA binding protein 1 (IGF2BP1/IMP1), an essential regulator of embryonic development that is expressed at low levels in most adult somatic cells (14–16). IMP1 is, however, re-expressed in some tumours, where it is linked to cancer cell invasiveness, and it is considered an important entry point to control tumour metastasis (16). Recent work by Huang and colleagues, has shown that IMP1, as well as its paralogues IMP2 and IMP3, recognises m6A-methylated RNA sequences, regulating the expression of *c-Myc* and other oncogenes in cancer cells (14). However, the molecular basis of m6A-based recognition by the IMP proteins is debated (1,17).

In this manuscript, we explore the molecular basis of m6A recognition by IMP1, the best studied of the three paralogues. We show that the protein interacts directly with the methylated targets forming a tight and long-living interaction and present a high-resolution solution structure for the protein–m6A complex. The structure reveals that IMP1 defines m6A-mediated target specificity very differently from the canonical YTH proteins, while the conservation of the amino acids dedicated to m6A recognition indicates that m6A recognition is a conserved protein function but also that is specific to this KH domain. Together, our results provide a first mechanistic understanding of recognition and indicate m6A methylation plays a different role in target selection by canonical and non-canonical regulators.

## MATERIALS AND METHODS

### Cloning

The KH34 di-domain cDNA construct (P387–A573, Y396F) of *G. gallus* insulin like growth factor 2 mRNA binding protein 1 (IMP1 – Gene ID 395953) and GDDG mutants (KH(3)4 and KH3(4)) were inserted into pETM11 expression vector as previously described (18,19). The V140I/P141S double mutant of KH(3)4 was generated using the Q5^®^ Site-Directed Mutagenesis Kit (NEB). Mutagenic primers (Sigma) were designed with the NEB changer webtool following kit specifications, and optimised using Primer3+ (20).

(V523I/P524S_F: GGTGGTGATTTCACGGGATCAGACCCCTGA; V523I/P524S_R: TCTGCAGCCGTCAGGTTCTGCAGCTCAT). Successful mutagenesis was confirmed by DNA sequencing (Source Bioscience).

### Protein expression and purification

All constructs were expressed as N-terminal 6xHis-tag fusion proteins in BL21(DE3) *Escherichia coli* cells (NEB). Unlabelled samples were obtained from protein expression in LB media, isotopic labelling was achieved by growing the cells in M9 minimal media supplemented with different combinations of ^15^NH_4_Cl, ^13^C-D-glucose and D_2_O as described (21,22). Cells were cultured at 37°C, and expression was induced overnight at 18°C by addition of 0.5 mM isopropyl β-d-1-thiogalactopyranoside. Cell pellets were resuspended in 10 mM Tris-HCl pH 8.0, 10 mM imidazole, 1 M NaCl, 5% (v/v) glycerol, 2 mM 2-mercaptoethanol, one cOmplete™ protease inhibitor cocktail tablet (Merck) per 50 ml of buffer, 0.01% Triton^TM^ X-100 (Sigma), 200 μg/ml lysozyme (Sigma), 0.01 mg/ml DNAse I (Sigma) and lysed by sonication. Proteins were purified from the soluble fraction by immobilised metal affinity chromatography (IMAC) using a HisTrap™ FF Nickel Sepharose Column (GE Healthcare), eluting with a linear gradient of 10 column volumes from 0 to 600 mM imidazole. The N-terminal 6xHis-tag was removed by overnight cleavage with 5 μM TEV protease at 4°C in 50 mM Tris-HCl pH 7.5, 150 mM NaCl, 2 mM 2-mercaptoethanol. Proteins were further purified by cation exchange on a HiLoad® SP Sepharose 26/10 column (GE Healthcare), eluted by applying a 0–100% gradient of 1 M NaCl in 10 mM Tris-HCl pH 7.3, 2 mM 2-mercaptoethanol. The eluted peak fractions were then applied to a HiLoad® 16/600 Superdex 75 pg column (GE Healthcare), equilibrated with 10 mM Na_2_HPO_4_ pH 6.5, 50 mM NaCl, 1 mM tris(2-carboxyethyl)phosphine) (TCEP). Peak fractions were concentrated to ∼20 mg/ml and purity assessed to be >95% using SDS-PAGE (23). Samples were snap frozen in small aliquots and stored at –80°C for use in further experiments. Protein concentration was determined from the absorbance at 280 nm using the theoretical extinction coefficient calculated by ProtParam ExPASy (24).

### RNA preparation

RNA oligonucleotides for NMR studies (5′-UCGGACU-3′ and 5′-UCGG(m6A)CU-3′) and BLI experiments (3′UTR Zipcode of β-actin mRNA—fragment 1229–1256 Gene ID NM_205518.2 – 5′-Bi-ACCGGACUGUUACCAACACCCACACCCC-3′ and 5′-Bi-ACCGG(m6A)CUGUUACCAACACCCACACCCC-3′) were purchased from Horizon Discovery Ltd. Prior to use, RNAs were deprotected following the manufacturer's instructions, lyophilised and resolubilized in D_2_O (Sigma-Aldrich). RNA concentrations were determined using UV/Vis absorption spectroscopy and extinction coefficients provided by the manufacturer.

### Nuclear magnetic resonance (NMR) spectroscopy experiments

^15^N-, ^15^N-^13^C- and ^2^H-^15^N-^13^C-labelled samples of the KH(3)KH4-UCGG(m6A)CU complex were prepared at a final concentration of 350 μM in NMR buffer (10 mM Na_2_HPO_4_ pH 6.5, 50 mM NaCl, 1 mM TCEP, 0.02% NaN_3_, 0.2 U/μl RNase inhibitor (ThermoFisher Scientific)), containing 10 or 99.8% D_2_O as appropriate. NMR spectroscopy experiments were performed on Bruker Avance spectrometers operating at 700, 800 and 950 MHz ^1^H frequency, processed using NMRpipe (25) and analysed with CcpNmr Analysis V2 (26).

Protein backbone resonance assignments were obtained from 2D ^1^H-^15^N HSQC, 2D ^1^H-^13^C HSQC (27), 3D HNCA, 3D HN(CO)CA, 3D HNCACB and 3D HN(CO)CACB spectra (28). Side-chain resonance assignments were determined from 3D H(CCO)NH, [^1^H-^13^C-^1^H] HCCH-TOCSY, [^13^C-^13^C-^1^H] HCCH-TOCSY (29), 3D ^15^N-NOESY-HSQC and 3D ^13^C-NOESY-HSQC (30) experiments. Resonance assignments of UCGG(m6A)CU RNA, free and in complex with KH(3)4, were obtained from 2D ^1^H-^1^H TOCSY, 2D ^1^H-^1^H NOESY spectra (27) either decoupled or un-decoupled. NOESY spectra were recorded using mixing times of 150 ms. TOCSY spectra were recorded using a mixing time of 60 ms.

Intramolecular NOEs were obtained from 3D ^15^N-NOESY-HSQC, 3D ^13^C-NOESY-HSQC experiments (30). Intermolecular NOEs were obtained from 2D ^1^H-^1^H NOESY (27), 3D ^15^N-NOESY-HSQC, 3D ^13^C-NOESY-HSQC (30), and 3D-filtered ^13^C-NOESY (31), with ^13^C and ^15^N rejected (150 ms mixing time) recorded on 1:2 protein (labelled):RNA (unlabelled) samples.

T1, T2 and {^1^H}-^15^N heteronuclear NOE relaxation experiments were recorded using the pulse sequences adapted from standard schemes (32) and analysed within CcpNmr Analysis V2 (26), by fitting the exponential decay to the peak volume over the course of the data collection. Where overlap in the signals prevented accurate measurements of peak volume, residues were excluded.

### NMR structure calculations

The structure of the KH(3)4-UCGG(m6A)CU complex was calculated using a semi-automated ARIA 2.3-based protocol (33), where distance restraints were input via integration of NOE cross-peaks obtained in 3D and 2D NOESY spectra using the XEASY program (34). The topology and parameter files for m6A were generated and optimized using the PRODRG Server (35).

Protein–protein NOE cross-peaks were calibrated automatically and iteratively assigned within ARIA, while peaks arising from RNA proton resonances were calibrated manually in a semi-quantitative fashion, as previously described (36). Protein angle restraints were obtained from CO, CA, CB, N and HN chemical shifts using TALOS (37). RNA angle restraints (α, ζ and δ) were obtained from ^1^H-^1^H TOCSY spectra and ^31^P-^1^H correlation spectra (36). Hydrogen bond restraints were added only in the final set of calculations and only in well-defined secondary structure elements if a proton was hydrogen-bonded in at least 50% of the initial set of structures. One hundred conformers of the KH(3)4-UCGG(m6A)CU complex were calculated with ARIA 2.3 (33) (iterations 0–7) and the 20 conformers with the lowest restraint energies were refined in a shell of explicit water. The 20 conformers with the lowest restraint energies, restraint violations and r.m.s. deviations from the ideal covalent geometry were taken as representative of the converged structures and selected for structural analysis. Structural statistics were computed for an ensemble of 20 deposited structures using PSVS 1.5 (38). A Ramachandran analysis of the structures show 89.0%, 10.1%, 0.9% and 0% of the protein residues in the most favoured, additional, generously allowed and disallowed regions, respectively.

All the structure images were generated with PyMOL Molecular Graphics System 2.0 (Schrödinger, LLC).

### NMR binding studies

NMR binding studies were conducted at 37°C on a Bruker Avance spectrometer operating at 800 MHz ^1^H frequency. For the titrations of the wild type and double mutant KH(3)4 protein, ^1^H-^15^N HSQC spectra were recorded on 80 μM protein samples with either UCGGACU or UCGG(m6A)CU added, at molar ratios of 1:0.5, 1:1, 1:2, 1:4, 1:8. The sequence-independent preference of KH4 for m6A vs A was determined with single-point titration ^1^H-^15^N HSQC experiments, by adding either NNNAN or NNN(m6A)N quasi-degenerate RNA pools into 80 μM protein samples in NMR buffer, to a protein-to-RNA molar ratio of 1:2.

NMR spectra were processed using TopSpin 4.0.6 (Bruker) and NMRPipe (25). Chemical shift perturbations (CSPs) of NH resonances in absence/presence of RNA were obtained by comparing ^1^H-^15^N HSQC spectra and calculated in CCPNMR Analysis (26) with the formula:


\begin{equation*}{\mathrm{CSP\ = \ }}\sqrt {{{\left( {{{\mathrm{\delta }}}_{{}_{}^{\mathrm{1}}{\mathrm{H}}}} \right)}}^{\mathrm{2}}{\mathrm{ + }}{{\left( {{\mathrm{0}}{\mathrm{.15}} \cdot {{\mathrm{\delta }}}_{{}_{}^{{\mathrm{15}}}{\mathrm{N}}}} \right)}}^{\mathrm{2}}} \end{equation*}

where δ_1H_ and δ_15N_ are the chemical shift differences of the ^1^H and ^15^N dimensions respectively.

### Biolayer interferometry (BLI)

BLI experiments were performed in 10 mM Na_2_HPO_4_ pH 6.5, 50 mM NaCl, 1 mM TCEP, 2 mg/ml bovine serum albumin (Sigma), 0.005% Tween-20 (Sigma-Aldrich), on an Octet Red 96 instruments (ForteBio, Inc. Menlo Park, CA) operating at 25°C. The assays were carried out in 96-well plates (ThermoFisher Scientific) and a sample volume of 280 μl. After pre-equilibration at 25°C, the 3′UTR Zipcode of β-actin mRNA (either methylated or non-methylated) was immobilised on streptavidin-coated biosensors (Sartorius) to a final concentration of 2 ng/μl and incubated with varying concentrations of KH34 (5–320 nM). *k*_obs_ values were extracted using the program Anabel (39). Association rate constants (*k*_on_) were determined from the slopes of plots of *k*_obs_ versus protein concentration. The values of the dissociation rate constant (*k*_off_) were instead determined using the single curve analysis method. *K*_D_ values were calculated as the ratios between *k*_off_ and *k*_on_. Experiments were performed in triplicate.

### Sequence alignments

Primary sequence alignments of *H. sapiens* and *G. gallus* IMP1 protein sequences were carried out with T-COFFEE multiple sequence alignment server (40) and alignment figures were generated using Jalview (41) using the CLUSTAL X conservation representation. The numbering reported in the whole manuscript is the one from *H. sapiens*, unless otherwise stated.

### Cell culture

HeLa cells were cultured in Dulbecco's modified Eagle's medium (DMEM) supplemented with 10% foetal bovine serum (FBS) and 1% penicillin-streptomycin, in a 5% CO_2_ incubator at 37°C. Cells were routinely tested for mycoplasma contamination.

Individual-nucleotide resolution UV-crosslinking and immunoprecipitation of protein–RNA complexes (iCLIP). iCLIP was performed using a previously reported protocol (42) (see also Supplementary Figure S10A) which was adapted as described below. Briefly, three HeLa cell biological replicates in 10 cm dish (80% confluency) were cross-linked at 150 mJ/cm^2^ in a Stratalinker 2400 at 254 nm and then lysed in 1 ml of 50 mM Tris-HCl, pH 7.4, 100 mM NaCl, 1% IGEPAL CA-630 (Sigma I8896), 0.1% SDS, 0.5% sodium deoxycholate. 0.8 U of RNase I (Thermo Scientific, EN0602) and 4 μl of Turbo DNase I (Ambion, AM223) were added to 1 mg (protein content) of lysate for RNA fragmentation and DNA digestion respectively. Samples were incubated with 5 μg of anti-IMP1 (Rabbit MBL, RN007P) or 5 μg of anti-IgG (Proteintech 30000-0-AP) antibodies, and coupled to Protein G beads (Dynal), rotating on a wheel at 4°C overnight. Pre-adenylated infrared dye-labelled IRL3 adaptor IRdye-800CW-DBCO (LI-COR, cat# 929-50000) (43) with sequence /5rApp/AG ATC GGA AGA GCG GTT CAG AAA AAA AAA AAA /iAzideN/AA AAA AAA AAA A/3Bio/ was ligated to RNA. The protein–RNA complexes were then separated by SDS-PAGE (23), blotted onto nitrocellulose membrane and visualised with an Odyssey scanning system (LI-COR). The desired region (determined from the RNAse gradient experiment in Supplementary Figure S10B) was excised from the membrane in small pieces and the RNA was released using proteinase K (Roche, 03115828001) digestion and incubation at 60 min at 50°C. For the RNase gradient experiment, 0.4, 0.8 or 2.5 U of RNase I (Thermo Scientific, EN0602) and 4 μl of Turbo DNase I (Ambion, AM223) were added to 1 mg (protein content) of lysate. Phenol–Chloroform extraction was performed to recover RNA. Reverse transcription was performed using Superscript IV Reverse Transcriptase (Life Technologies) and primers containing Unique Molecular Identifiers and barcodes (XXXXX) to allow multiplexing: /5Phos/ WWW *XXXXX* NNNN AGATCGGAAGAGCGTCGTGAT /iSp18/ GGATCC /iSp18/ TACTGAACCGC. cDNA molecules were purified using AMPure XP beads (Beckman Coulter, USA), circularised using Circligase II (Epicenter), and purified using AMPure XP beads (Beckman). After PCR amplification, libraries were size-selected by gel purification and size distribution was assessed using a2400 Bioanalyser (Agilent). QuBit dsDNA HS Assay (ThermoFisher Scientifics) was used to quantify libraries. The same quantity of cDNA for each sample in the library was sequenced as single end 100 bp reads on Illumina HiSeq 4000.

### Processing of iCLIP data

iCLIP reads from the HeLa samples and IgG control were processed using the iMaps webserver (https://imaps.genialis.com/iclip), with the following steps: demultiplexing using sequencing barcodes, UMI identification, adapter trimming, pre-mapping to rRNAs and tRNAs, alignment to genome using Spliced Transcripts Alignment to a Reference (STAR) (44), cross-link sites assignment, peak calling using Paraclu (45). Significant cross-link sites were identified using the ‘iCount peaks’ tool, while peaks were defined by clustering the significant cross-link sites using default parameters. Summary files based on cross-link events on gene type, biotype and gene region were generated. The percentage of total counts for each genomic region was calculated using the cross-link counts obtained from the merged replicates and normalised to each genomic length. FastQC 0.11.5, https://www.bioinformatics.babraham.ac.uk/projects/fastqc/, PCR duplication ratio, quality of sequencing and alignment statistics were performed on each individual samples. All samples showed a high number of uniquely mapped reads (on average 2.2 × 10^6^) with low PCR duplicate ratio (1.57–1.74). Cross-link or peak bed files from replicates were merged using the iMaps group function. Peaks were then called using iMAPS default parameters, and the output bed/bedgraph files were used for further analysis. The human GRCh38 genome build and GENCODE version 36 annotation were used. Correlation between replicates was assessed using the multibamSummary function from DeepTools v3.5.5, with default parameters. Scatterplots were generated with the plotCorrelation function using Spearman method and the option removeOutliers (46).

### Visualisation of iCLIP tracks

HeLa iCLIP cross-link sites and m6A-sites obtained from a publicly available miCLIP dataset (47) (option -y) were visualized using Clipplotr v1.0.0 (48). iCLIP signals were normalised on library size and scaled to cross-links per million. Gaussian smoothing with a sliding window of 100 nucleotides was used. Plot size was modified to be 100 mm high and 200 mm wide.

### Re-analysis of published datasets

Publicly available data from IMP1 CLIP (GSE78509) (49) and m6A-CLIP (GSM2300426) (47) experiments were obtained from the GEO database.

For the m6A reference file, the raw BED files containing nucleoplasmic and cytoplasmic m6A sites were downloaded from the GEO database. The BED files were lifted to their equivalent ones in the hg38 reference genome using the LiftOver tool of the UCSC Genome Browser (http://genome.ucsc.edu) (50). Common entries within the cytoplasmic and nucleoplasmic datasets were selected using the bedtools intersect command (51), and annotated using the gencode.v36.annotation.gtf file for gene only downloaded from https://www.gencodegenes.org/human/release_36.html and filtered for remaining duplicates by removing entries with identical start and end coordinates.

For the IMP1 iCLIP, the raw BED files for each replicate were also download from the GEO database and the BED files were lifted to their equivalent ones, annotated and filtered as detailed above for the m6A reference file. Common genes were then compared to our IMP1 iCLIP data using the bedtools intersect command (51).

### Calculation of the bound ACTB zipcode RNA

For a simple competition equilibrium, the amount of (m6A methylated) ATCB RNA Zipcode bound to IMP1, can be calculated by first obtaining the amount of free unbound protein from the solution to the following equation (52):


\begin{eqnarray*} {[{\mathrm{P}}]}^{\mathrm{3}}{\mathrm{ + }}{[{\mathrm{P}}]}^{\mathrm{2}}\left( { - {{[{\mathrm{P}}]}}_{\mathrm{t}}{\mathrm{ + K}}_{\mathrm{D}}^{{\mathrm{RNA}}}{\mathrm{ + K}}_{\mathrm{D}}^{{\mathrm{meRNA}}}{\mathrm{ + }}{{\left[ {{\mathrm{RNA}}} \right]}}_{\mathrm{t}}{\mathrm{ + }}{{[{\mathrm{meRNA}}]}}_{\mathrm{t}}} \right)\\ {\mathrm{ + }}\left[ {\mathrm{P}} \right]( - {[{\mathrm{P}}]}_{\mathrm{t}}{\mathrm{K}}_{\mathrm{D}}^{{\mathrm{RNA}}} - {[{\mathrm{P}}]}_{\mathrm{t}}{\mathrm{K}}_{\mathrm{D}}^{{\mathrm{meRNA}}}{\mathrm{ + K}}_{\mathrm{D}}^{{\mathrm{RNA}}}{\mathrm{K}}_{\mathrm{D}}^{{\mathrm{meRNA}}}\\ {\mathrm{ + }}{\left[ {{\mathrm{RNA}}} \right]}_{\mathrm{t}}{\mathrm{K}}_{\mathrm{D}}^{{\mathrm{meRNA}}}{\mathrm{ + }}\left[ {{\mathrm{meRNA}}} \right]{\mathrm{K}}_{\mathrm{D}}^{{\mathrm{RNA}}}) - {[{\mathrm{P}}]}_{\mathrm{t}}{\mathrm{K}}_{\mathrm{D}}^{{\mathrm{RNA}}}{\mathrm{K}}_{\mathrm{D}}^{{\mathrm{meRNA}}}{\mathrm{ = 0}}\end{eqnarray*}

where [P]_t,_ [RNA]_t_, and [meRNA]_t_ are the total concentrations of IMP1, unmethylated RNA and methylated RNA, and ${\mathrm{K}}_{\mathrm{D}}^{RNA}$ and ${\mathrm{K}}_{\mathrm{D}}^{meRNA}$ are the equilibrium dissociation constants of the two reactions:


\begin{equation*}{\mathrm{P}} + {\mathrm{RNA}} \mathbin{\lower.3ex\hbox{$\buildrel\textstyle\rightarrow\over {\smash{\leftarrow}\vphantom{_{\vbox to.5ex{\vss}}}}$}} {\mathrm{\ P}} . {\mathrm{RNA}}\end{equation*}

\begin{equation*}{\mathrm{P}} + {\mathrm{meRNA}} \mathbin{\lower.3ex\hbox{$\buildrel\textstyle\rightarrow\over {\smash{\leftarrow}\vphantom{_{\vbox to.5ex{\vss}}}}$}} {\mathrm{P}}.{\mathrm{meRNA}} \end{equation*}

The concentrations of bound unmodified and methylated RNA, [P.RNA] and [P.meRNA] are then calculated as it follows:


\begin{equation*}{\mathrm{[P . RNA]\ = }}\frac{{{{{\mathrm{[RNA]}}}}_{\mathrm{t}}{\mathrm{[P]}}}}{{\left( {{\mathrm{K}}_{\mathrm{D}}^{RNA{\mathrm{\ }}}{\mathrm{ + [P]}}} \right)}}\ \end{equation*}

\begin{equation*}{\mathrm{[P . meRNA]\ = }}\frac{{{{{\mathrm{[meRNA]}}}}_{\mathrm{t}}{\mathrm{[P]}}}}{{\left( {{\mathrm{K}}_{\mathrm{D}}^{meRNA{\mathrm{\ }}}{\mathrm{ + [P]}}} \right)}}\ \end{equation*}

### Data analysis and display

Data were analysed and displayed using Microsoft Excel, Prism 7 GraphPad (Dotmatics), RStudio (v 4.1.2) and Biorender.com.

## RESULTS

### IMP1 KH4 recognises directly the m6a methylation of the cognate sequence

**Figure 1.**
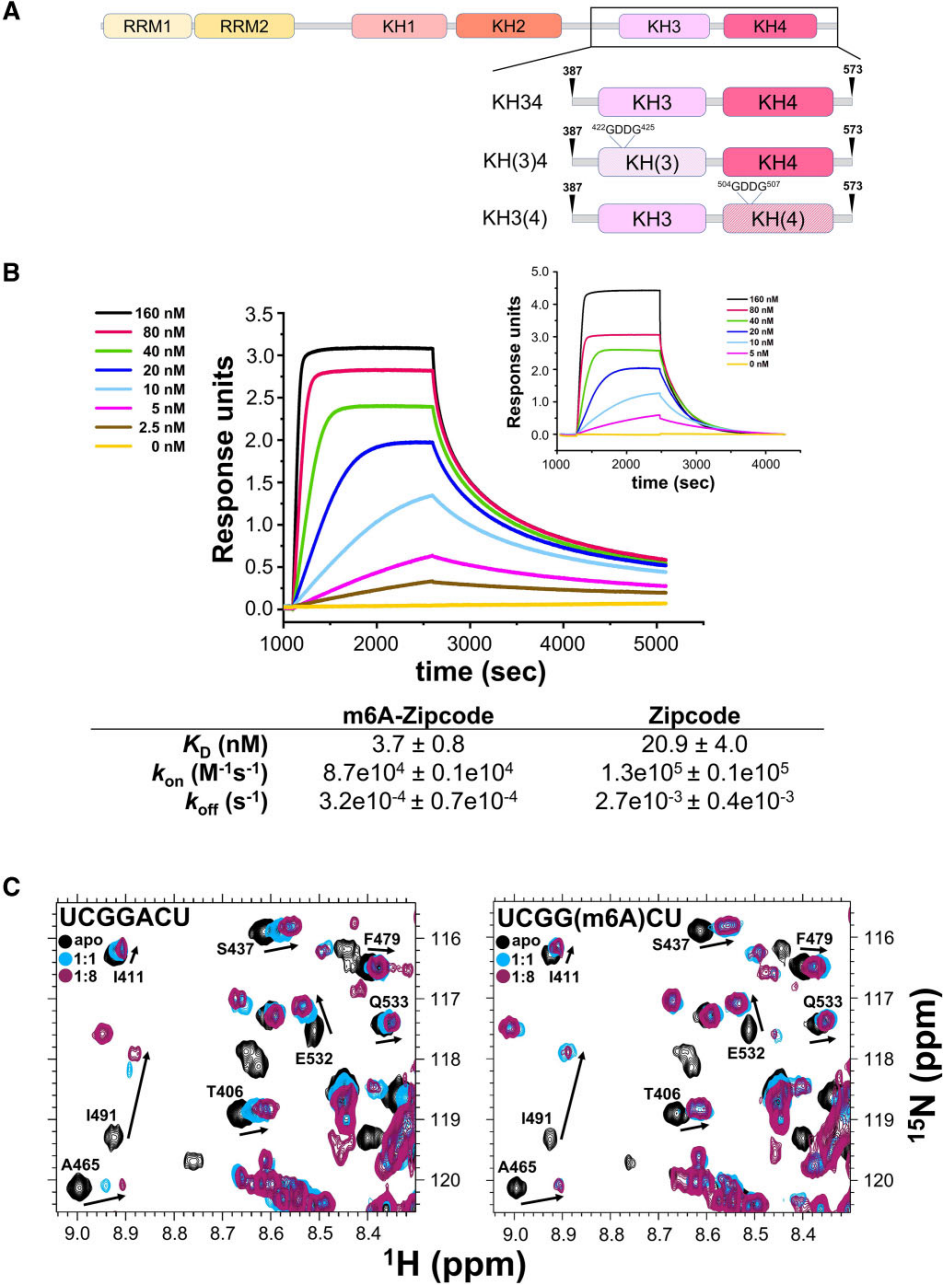
m6A methylation stabilizes the IMP1−RNA interaction. (**A**) IMP1 domain structure organisation. Arrowheads indicate boundaries of the recombinant IMP1 KH34 constructs used in this study. The *H. sapiens* numbering is reported and used throughout, unless otherwise specified. The positions of the GDDG mutations of the GxxG loops used to obtain the KH(3)4 and KH3(4) constructs are indicated (**B**) *G. gallus* IMP1 KH34−m6A Zipcode RNA binding measured by BLI. The left panel shows the interferograms obtained by addition of increasing concentrations of IMP1 to immobilized methylated Zipcode RNA. The right panel reports the equivalent interferograms previously obtained with un-methylated Zipcode RNA KH34 (19). In these plots, the interference change during the experiment is expressed as response units, and reports on the protein's association and dissociation to the immobilised RNA at different IMP1 concentrations. An example analysis of the kinetic data is shown in Supplementary Figure S1. (**C**) ^1^H-^15^N HSQC NMR titrations of IMP1 KH(3)4. The protein was titrated with either UCGGACU or UCGG(m6A)CU RNA, comprising the minimal cognate sequence augmented by one U both 5′ and 3′ to avoid any boundary effects. A well-dispersed, representative region is displayed, full spectra are shown in Supplementary Figure S2. Changes in peak positions report on the fraction of bound protein.

IMP1 contains six single-stranded RNA-binding domains, two RNA recognition motifs (RRM) and four K homology domains (KH) organized in three di-domain structural units, RRM12, KH12 and KH34 (15,17) (Figure 1A). Huang and co-workers have reported that the KH34 di-domain is the key element for m6A recognition by IMP proteins and proposed that direct methyl recognition mediates the selection of cancer-related mRNAs in the cell (14). However, the m6A recognition mode of IMP proteins is debated, as a later study instead reported that IMP3 is not likely to recognize the methyl group directly (53). Here, we have explored how IMP1 KH34 recognises the m6A-methylated β-actin (ACTB) Zipcode RNA, a well-characterized target that has been previously used to dissect IMP1 recognition of non-methylated RNA (19). Our biolayer interferometry (BLI) experiments show that IMP1 KH34 directly recognizes the m6A methylation of the RNA. While m6A does not significantly affect the IMP1−RNA association kinetics (*k*_on_), it increases the lifetime (1/*k*_off_) of the protein−RNA complex by about 8-fold (Figure 1B; Supplementary Figure S1), which translates to an overall five-fold increase in affinity. Importantly, these kinetic data indicate that IMP1 possess an intrinsic m6A selectivity that depends on the methyl group increasing the stability of the complex.

Notably, we and others have previously shown that the IMP1 KH4 domain recognises a GGAC RAC-like sequence (19,54), and this domain is therefore most likely to mediate IMP1 recognition of m6A methylated RNAs. We examined KH4-methyl recognition using a previously tested KH34 di-domain construct where KH3-RNA binding is knocked out using a double DD mutation, KH(3)4, in the conserved RNA-binding GxxG motif (18) (Figure 1A). Using nuclear magnetic resonance (NMR), we compared the interaction of this construct with the m6A-methylated and un-modified KH4 recognition sequence in the ACTB mRNA Zipcode element. The magnitude of the RNA-induced chemical shift changes we observe, indicate that the protein binds the m6A-RNA stoichiometrically, but has a lower affinity for the unmodified RNA. These data confirm that the KH4 domain directly recognizes the methylation of the target sequence (Figure 1C; Supplementary Figure S2) and validate our BLI-based analysis using an orthogonal technique.

### m6A recognition is mediated by a dedicated and conserved structural element

**Figure 2.**
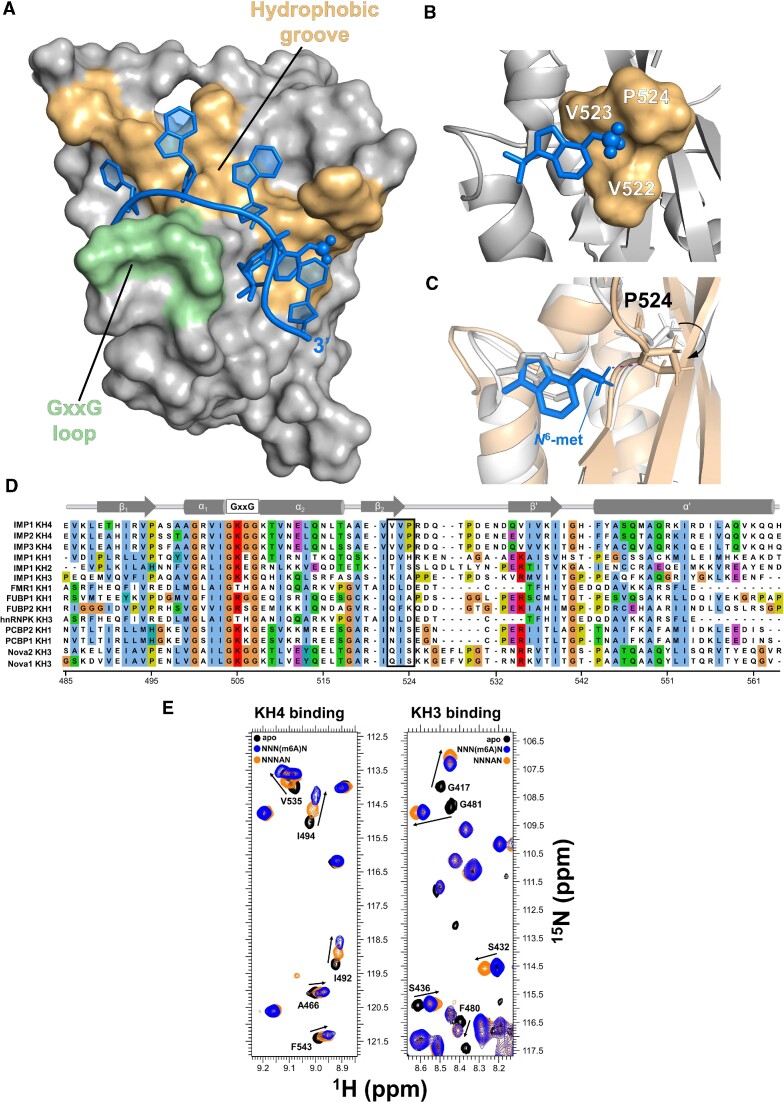
The m6A nucleobase interacts with a hydrophobic platform. (**A**) Surface representation of KH(3)4−UCGG(m6A)CU complex (PDB code 8COO). The physiological five-nucleotide core RNA sequence (CGGAC) is displayed. The RNA is shown in blue sticks, with the *N*^6^-methyl-group of m6A represented as spheres. The GxxG loop and the hydrophobic groove of KH4 are coloured in light green and wheat respectively. The family of converged structures is shown in Supplementary Figure S3A–C, details of the NOEs that define the interaction are shown in Supplementary Figure S3D. (**B**) The ‘hydrophobic cradle’. The surface representation of the three ^522^VVP^524^ residues forming the hydrophobic cradle is displayed in wheat while the *N*^6^-methyl-adenine of m6A is shown in blue sticks, with the methyl group in spheres. (**C**) Conformational change of P524 upon m6A-RNA binding. The aligned KH(3)4–UCGG(m6A)CU and –UCGGACU complexes are depicted in wheat and grey respectively, with the distance between Hα of P524 and the *N*^6^-methyl-group of m6A (blue) highlighted with purple dash. (**D**) Sequence alignment of KH domains. CLUSTAL X alignment of KH domains from a range of human RNA-binding proteins. A cartoon of the KH-domain canonical secondary structure is shown above the alignment. The black box highlights the three IMPs KH4 residues involved in the formation of the ‘hydrophobic cradle’. Abbreviations: FMR1: fragile X messenger ribonucleoprotein 1; FUBP: far upstream element binding protein 1; hnRNPK: heterogeneous nuclear ribonucleoprotein K; PCBP: poly (rC) binding protein; Nova: NOVA alternative splicing regulator. The *H. sapiens* numbering is reported at the bottom of the alignment. (**E**) Single-point ^1^H-^15^N HSQC NMR titrations of KH(3)4 and KH3(4) with quasi-degenerate pools NNNAN and NNN(m6A)N. A representative region is displayed, while the full spectra are reported in Supplementary Figure S7. The KH4 domain recognises m6A independently of the flanking sequence, while KH3 prefers un-modified RNA targets.

KH domain recognition of specific sequences is mediated by the recognition of the shape and moieties of the RNA bases in the domain's hydrophobic groove (55). As it is difficult to predict how such a groove may recognise a methylated nucleobase, we determined the structure of the KH(3)4−m6A RNA complex using well-established solution NMR methodologies (19,56). In the structure, the m6A methylated RNA binds KH4 using the canonical KH RNA binding surface, where the RNA backbone interacts with the conserved GxxG loop and the nucleobases make contacts with the protein's hydrophobic groove (Figure 2A; Supplementary Figure S3A-D; Supplementary Table S1). The m6A nucleobase is precisely positioned by several nuclear Overhauser effect (NOE)-based distance correlations between the methyl group and nearby amino acids (Supplementary Figure S4). The adenine nucleobase rests on a hydrophobic platform built by the sidechains of two consecutive valine residues, V522 and V523 (Figure 2B; Supplementary Figure S4) and extended by a proline, P524, that rotates to assemble a shallow ‘hydrophobic cradle’ to accommodate the methyl group (Figure 2C). Notably, the hydrophobic recognition of m6A requires a relatively minor rearrangement of the adenine position from that observed in the complex with un-modified RNA (19) (Figure 2C: Supplementary Figure S3E). Sequence comparison of IMP1 and its two paralogues, indicate that the Val-Val-Pro residues involved in the binding of m6A are phylogenetically conserved in the IMP family (Figure 2D; Supplementary Figure S5). Notably, the first valine of the triad, valine 522 in IMP1, corresponds to an isoleucine in IMP2, but the Cγ1-methyl of Val and Cγ2-methyl of Ile can be substituted without significant disruption of the hydrophobic cradle. Importantly, these residues are not present amongst other KH domains (Figure 2D; Supplementary Figure S5), consistent with a common mechanism of direct m6A recognition by IMP1, IMP2 and IMP3. Moreover, despite changes in backbone conformation through movement of the proline, and some smaller changes in the contacts made by other nucleotides (Supplementary Figure S3D, E), the dynamics of the RNA−protein complex in this region of the protein are not substantially changed by methylation (Supplementary Figure S6). Given the hydrophobic nature of the interaction, we expect that the discrimination is entropy-driven and likely mediated by the displacement of ordered water molecules making contacts with the methyl group of the adenine; notably, the entropy of dehydration is an important component in KH-mediated nucleic acid recognition (57). Together, our analysis indicates that m6A recognition is mediated by a local structural rearrangement that creates a shallow hydrophobic cradle to accommodate the m6A methyl group.

The structure of the IMP1−m6A-RNA complex indicates that the recognition of the methylated RNA is likely to be driven by the local interactions made by the m6A methyl group. In order to verify that the recognition does not depend on a specific underlying sequence, we compared the binding of IMP1 KH(3)4 to the m6A and A nucleotides in the context of randomised RNA. The larger RNA-induced chemical shift changes we observe in our NMR spectra for the methylated RNA, indicate that the preference for m6A is maintained independently of the host sequence (Figure 2E; Supplementary Figure S7). Interestingly, a similar experiment using a KH34 protein where only KH3 binds RNA, KH3(4) (Figures 1A and 2E; Supplementary Figure S7), showed that KH3 prefers A to m6A, and confirmed that the ability of IMP1 to recognize m6A is specific to KH4.

**Figure 3.**
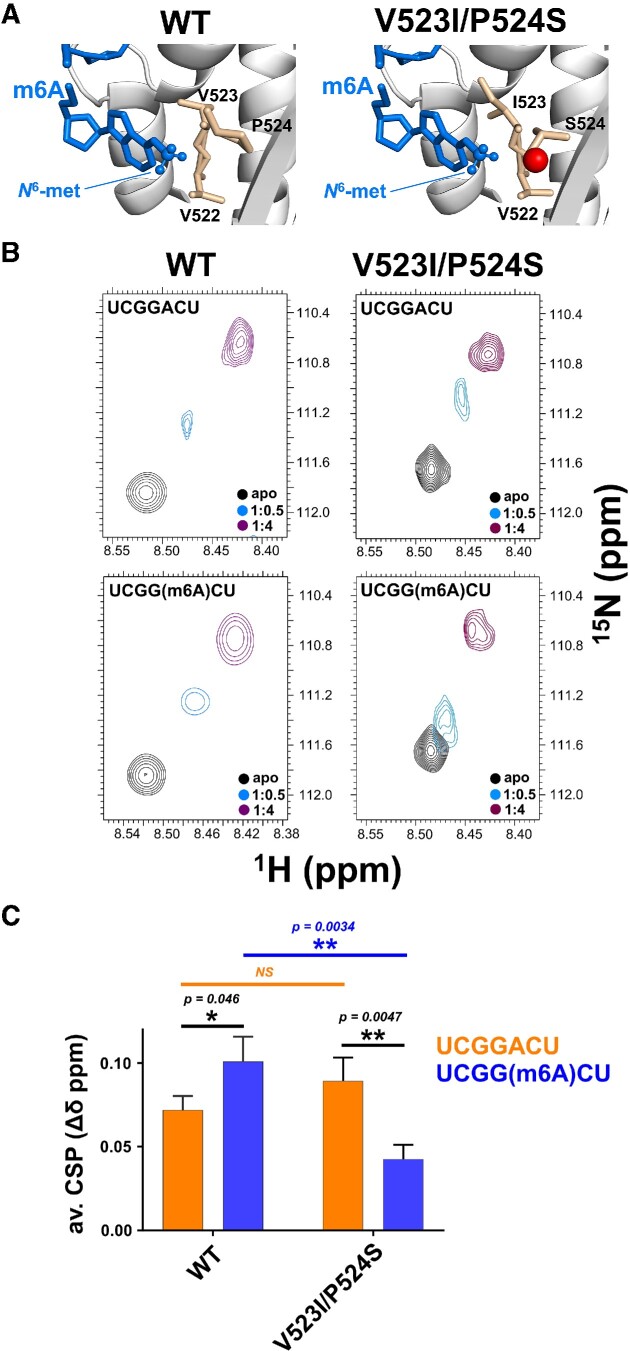
m6A recognition is mediated by two conserved amino acids. (**A**) Position of mutated amino acids on the KH4–m6A RNA structure. The m6A group is displayed using blue sticks, with the *N*^6^-methyl-group represented as spheres. The residues of the hydrophobic cradle are shown in wheat sticks, with the polar serine oxygen displayed as a red sphere. (**B**) Characterisation of the V523I/P524S double mutant. ^1^H-^15^N HSQC NMR titrations of IMP1 KH(3)4 and the double mutant V523I/P524S with UCGGACU and UCGG(m6A)CU RNAs. The amide peak of S545 is displayed as a reporter. The full spectra of the titrations are reported in Supplementary Figure S9A. **(C)** Quantification of chemical shift perturbations (CSP) of WT and V523I/P524S double mutant upon binding to the two RNAs. The quantification presented is the weighted mean ± weighted SD of the largest (11) shifts reported in Supplementary Figure S9B. Statistical significance was calculated with a homoscedastic Student's *t*-test with * indicating *p* ≤ 0.05 and ** indicating 0.05 < *p* ≤ 0.005.

Finally, to validate the mechanism of m6A recognition discussed above, we tested whether mutation of the two key residues of the hydrophobic cradle (V523I/P524S) may eliminate the m6A-RNA specificity. The mutations were designed to change the local protein packing and disrupt the hydrophobic interaction between the conserved P524 and the RNA methyl group (Figure 3A). Together, this double mutation was designed to achieve better packing without changing the structure of the protein. We probed the structure and RNA binding properties of the double mutant using ^1^H-^15^N-correlation 2D NMR experiments, which indicated that the mutation does not significantly change the structure of the di-domain (Supplementary Figure S8). Next, we compared the changes in the spectra of wild type and mutant KH(3)4 proteins upon binding to either un-methylated RNA or m6A-RNA. The RNA-dependent changes in peak positions show that, while the affinity for the target sequence is not substantially changed by the mutation, the mutant affinity for the methylated RNA is significantly decreased (Figure 3B and C; Supplementary Figure S9). That is, the double mutation reverses the m6A specificity of the KH4 domain and confirms the key role of the residues engaged in the formation of the hydrophobic cradle. Notably, the double mutant also represents a tool for the community to directly probe the m6A-dependency of IMP1−RNA interactions and the regulatory role of m6A in target selection.

### A new concept for m6A-mediated IMP1 target selection

**Figure 4.**
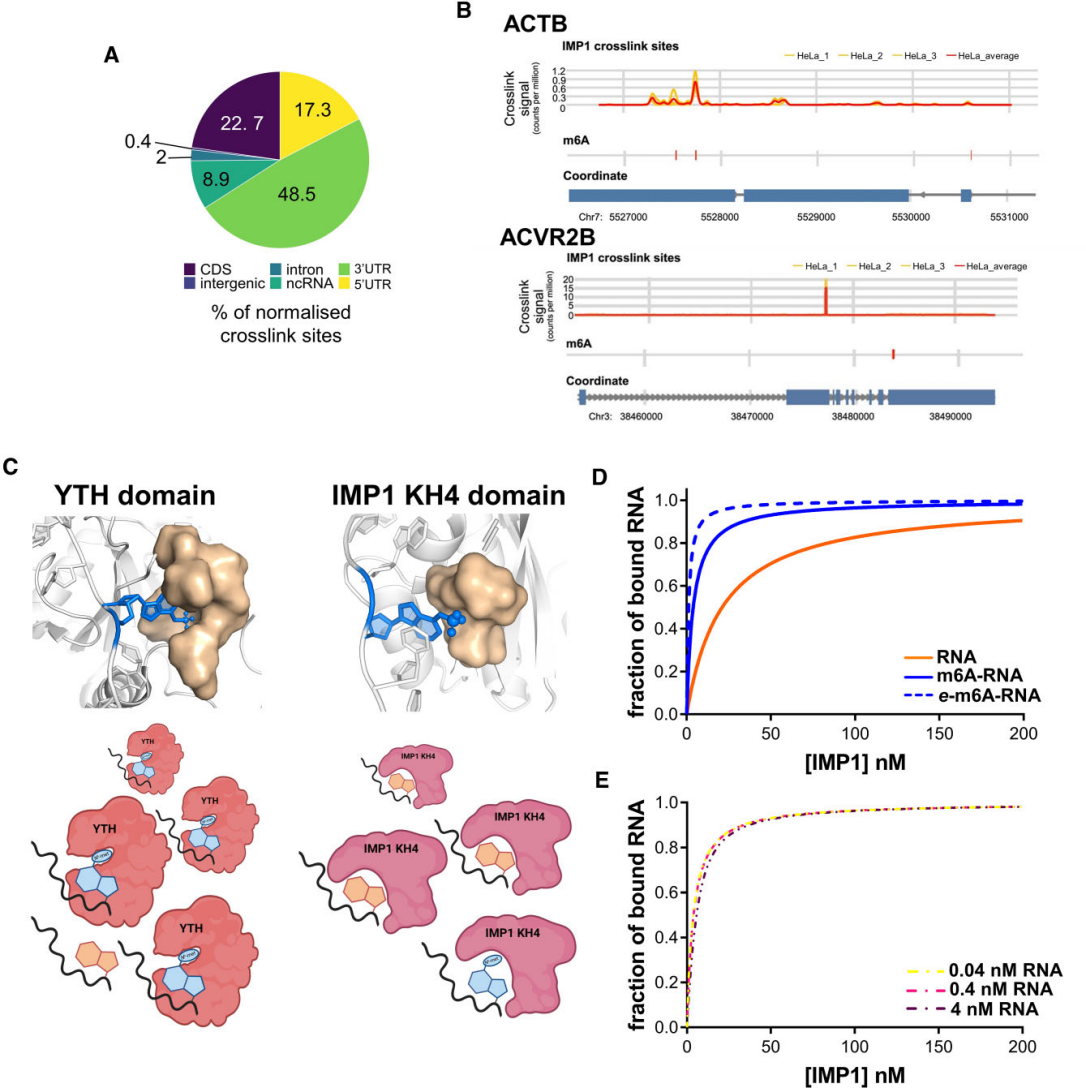
IMP1 recognises and binds both m6A-methylated and non-methylated RNA. (**A**) Percentage of unique cDNAs mapping to each transcriptomic region. Counts were normalised to length and percentage of total counts was calculated. (**B**) Representative iCLIP and miCLIP tracks of the ACTB and activin receptor type-2B (ACVR2B) mRNAs. For the iCLIP tracks the signals for each HeLa replicate are shown in yellow and the merged signal from all three replicates in red. On the miCLIP tracks, a red line shows the presence of a detected m6A-site. Experimental controls and the tracks of additional IMP1 targets are reported in Supplementary Figure S10. (**C**) Comparison of target recognition by the two m6A-RNA readers YTH and IMP1 KH4 domains—working model. While YTH forms a deep cleft where the m6A is trapped by the ‘hydrophobic cage’, KH4 of IMP1 provides a shallower hydrophobic cavity. The surface of the three tryptophan residues that create the YTH cleft and the amino acids and of the IMP1 KH4 Val-Val-Pro sequence are displayed and coloured in wheat. PDB codes used: 8COO, 2MTV (7). (**D**) Fraction of ACTB RNA Zipcode bound to IMP1 as a function of IMP1 concentration, at *K*_D_ values of 20.9 nM (*K*_D_ measured for the un-modified RNA, orange), 3.7 nM (K_D_ measured for m6A RNA, blue, continuous, line) and 1 nM (a lower *K*_D_ to highlight the decreasing effect of higher affinity, blue, dashed line—e-m6A-RNA). The total concentration of ACTB RNA Zipcode, used in these calculations is 0.4 nM. The fraction is calculated using a simple competition equilibrium model, and methylation yields an increase of several-fold at 5 nM concentration of free protein but only a marginal one at a concentration of 25 nM. (**E**) Fraction of ACTB RNA Zipcode bound to IMP1 as a function of IMP1 concentration at a *K*_D_ of 3.7 nM and RNA concentrations of 0.4 nM (purple—physiological), 4 nM (pink) and 0.04 nM (yellow).

Our structural data show that, as for YTH reader proteins, IMP1 recognises directly the m6A nucleotide. Next, as the level of m6A selectivity of the two proteins is quite different (7), we assessed whether the YTH proteins’ regulatory model, where m6A ubiquitously regulates the selection of the RNA targets, can also be applied to IMP1. We performed a transcriptome-wide analysis of IMP1−RNA interaction in HeLa cells using iCLIP and examined whether IMP1 binding peaks superimpose with known sites of m6A methylation (Supplementary Figure S10A–G). A first analysis of our iCLIP data indicated that IMP1 interacts mainly in the 3′UTR of the mRNA, as previously reported (Figure 4A), and comparison of the bound RNAs with those from a published IMP1 iCLIP dataset obtained in a different cell line (49), showed that nearly half of the IMP1-bound transcripts in the two cell types are the same (Supplementary Figure S10F), confirming the expected IMP1 binding landscape. An analysis of IMP1 binding and methylation in the ACTB mRNA transcript both confirmed that IMP1 interacts with the Zipcode recognition element, but also that the GGAC sequence targeted by KH4 superimposes with a site of methylation (47) (Figure 4B). However, further examination of the data shows that, while IMP1 peaks localize on m6A sites in ACTB and other well-studied IMP1 mRNA targets, similar to what previously described for cancer targets, that is not the case for many other transcripts (Figure 4B; Supplementary Figure S10G). This analysis implies that, in contrast to what is observed for YTH domains, m6A does not represent a ubiquitous layer of regulation in IMP1−RNA target selection. To provide a molecular rationale for these differences, we compared the structural features of m6A recognition by YTH and IMP proteins. In YTH domain-m6A RNA recognition, the m6A is positioned in a deep cleft, and the methyl group is inserted into a hydrophobic cage created by three conserved tryptophan residues (6,7,12) (Figure 4C). These solvent-excluded intermolecular contacts increase YTH affinity for m6A-methylated RNA by 1–2 orders of magnitude (6,7). In contrast, IMP1 directly contacts the m6A nucleotide using a more open hydrophobic platform, where the methyl group remains partially solvent exposed. This is consistent with the more modest m6A-mediated increase in affinity. Together, this suggests a concept for non-canonical m6A readers where, unlike what is observed for YTH proteins, methyl regulation represents a nuanced mechanism of recognition selective for defined cellular contexts and targets.

To better rationalize, at the mechanistic level, how the impact of the methylation-driven increase in IMP1 affinity may vary depending on the physiological (and pathological) changes in protein and RNA abundance, we performed a set of model calculations using a straightforward competition equilibrium model to describe the IMP1−Zipcode RNA interaction at a range of protein and ACTB mRNA concentrations around the reported cellular values (19,58,59). Our results highlight that, at low nanomolar protein concentration, the *K*_D_ differential means methylation leads to a significant increase in selectivity of m6A-RNA over unmodified RNA (Figure 4D). Importantly, the effect is much more pronounced at an IMP1 concentration of 5 nM (3-fold) than at 25 nM (∼25%), highlighting that the system needs to be well poised to achieve an efficient discrimination. Notably, an additional calculation of IMP1 binding at 1 nM *K*_D_ allowed us to visualize how a further hypothetical increase in affinity would yield diminishing returns in terms of RNA binding (Figure 4D). While the effect of methylation on complex formation strongly depends on the protein concentration, at the very low reported cellular RNA concentration, RNA dependence of complex formation is minimal (Figure 4E). Virtually identical binding trends can be calculated for ACTB mRNA at concentrations of 0.4 nM (the reported cellular concentration (19,58)), 0.04 and 4 nM. This illustrates the vanishing effect of changes in RNA concentration, but also indicates that the methylation may regulate IMP1 binding to both low and high abundance RNA targets in a similar fashion.

Notably, like many other RNA binding proteins, IMP1 has been reported to bind to thousands of target sites (16,49), and only a share of the total pool of protein can therefore be considered as free protein. It is difficult to place a precise value on this share, which is also expected to vary at different times and in different cell types. Interestingly, a ratio of ∼1/10^3^ is observed between the count of unique cDNAs mapping to the ACTB Zipcode peak (116) and the count of cDNAs mapping to all IMP1 peaks in our iCLIP study (∼160000). cDNAs count does not quantitatively translate to occupancy, but we reasoned this broad comparison indicates that a large proportion of the IMP1 protein is likely to be bound to the RNA targets. That is, the IMP1−m6A ACTB interaction discussed in this study suggests that the presence of a large number of competing RNA target sites reduces the effective concentration of IMP1 to the range where m6A regulation on RNA binding is strongest, i.e., in the intermediate-to-low nanomolar range. This a concept that could be explored by the community beyond the effect of methylation, perhaps with the help of recent variants of the iCLIP method that report more directly on the protein occupancy of the RNA targets (e.g. (60)).

## DISCUSSION

The recognition of methylated RNA by m6A readers is an essential step to translate the m6A signal into regulatory outputs. While m6A recognition by YTH proteins is well characterised, a largely unanswered question in the field is how RNA-binding proteins that do not include a YTH domain recognise methylation sites. Understanding the molecular basis of this recognition is important to inform our thinking on how the interaction may be regulated during development and disease. In this study, we have described the molecular basis of IMP1−m6A recognition and its regulation and discussed the implications for the selection of the cellular targets. A first question we asked is whether IMP1 and its paralogues directly recognise m6A methylated RNA. Our analysis demonstrated that recognition of the m6A is indeed direct and takes place through a conserved hydrophobic element. A mutational analysis confirmed that this element is dedicated specifically to the recognition of the m6A methyl group, rather than contributing to RNA sequence specificity or shaping/stabilizing protein structure. Importantly, the conservation of the m6A-interacting residues in the IMP1 family and across evolution suggests that m6A regulation is important to the physiological function of these proteins. This is useful, as non-canonical regulators have been examined mostly in disease and non-physiological settings, and their m6A-mediated role in physiological processes remains largely unresolved (61).

Importantly, while YTH proteins have been shown to require m6A methylation for RNA target recognition, we show here that IMP1 can recognise specific unmodified RNA sequences with high affinity and select both m6A and un-modified targets in the cell. We therefore propose that m6A mediates a nuanced regulation of the IMP1−RNA interaction whose effect is target-specific and that depends on protein concentration and cellular settings. This concept helps to rationalize how IMP1, and other multi-functional RNA regulators, can integrate epitranscriptomic information into their networks. It also helps to explain the differences between the RNA binding landscape of YTH, which largely follows the sites of m6A methylation, and one of IMP1, which is more complex and where only a fraction of the binding sites is methylated.

In the context of this concept, it is then interesting to consider whether m6A regulation may be restricted to specific m6A-hosting sequences. While we did not explore this in detail, we show that m6A selectivity applies in the context of randomised RNA sequence. It seems therefore unlikely that recognition is strongly associated with a specific version of the DRACH sequence—beyond the general sequence requirements of IMP1. This reinforces a model where the selectivity of m6A regulation of IMP1 likely depends on multiple variables, and, rather than providing an on/off molecular switch, enhances target selectivity in specific cellular settings. Consistent with this hypothesis, while finalizing this work, a manuscript was published proposing that, as for other m6A regulators, the *in cell* structure of the RNA may also play a role in the interaction (62).

At a mechanistic level, a modelling calculation indicates that, m6A methylation is most effective at low protein concentration and largely independent from the concentration of RNA. IMP1 concentration also varies very significantly during development, and it is an important factor in tumour cell invasiveness. In this context, changes in the protein concentration are, in principle, an effective tool to tune the effect of m6A. Notably, an equivalent calculation (Supplementary Figure S11) employing the published data on the m6A/A binding affinity of YTH domain(s) suggests that in contrast to IMP1, YTH proteins can effectively discriminate *N*^6^-methylated from non-methylated RNA at sub–micromolar protein concentration. Importantly, direct recognition of the m6A group implies that IMP1 may compete for a specific m6A signal with YTH and any other readers able to bind the m6A group. While a detailed investigation of the relationship between different readers is not in the scope of this paper, it is pertinent to mention that both the affinity of the YTH domains (6,7) and the cellular concentration of YTHDF2 (63), are significantly lower than those of IMP1, and that this protein would not outcompete IMP1 based on binding affinity and concentration only.

Finally, the dedicated IMP1 m6A recognition element is conserved amongst the paralogues of the IMP family, which, like IMP1, are regulated by m6A in highly proliferating cells (14). Although differences due to expression levels and to the sequence specificity of individual domains exist, the proteins of this class share a physical framework for m6A recognition. Looking more broadly, non-YTH RNA regulators, are a heterogeneous group of proteins that do not share the IMP1 recognition element, and it seems likely that the details of recognition would vary. However, our study highlights that the discrimination of m6A methylated RNA can be developed by adding one or two amino acids to an existing hydrophobic surface. In most of the reported studies of m6A recognition by non-YTH reader proteins, the level of discrimination reported is, similarly to IMP1, a few-fold and seems possible that m6A discrimination is achieved in a range of RNA binding folds making use of minimal structural specializations.

## Supplementary Material

gkad534_Supplemental_File

## Data Availability

The accession number for the atomic coordinates of the NMR structure ensemble and the corresponding chemical shift assignment are deposited in the Protein Data Bank, accession No 8COO. The iCLIP data generated for this paper have been deposited in NCBI Gene Expression Omnibus (GEO) under accession number GSE214367. According to UK Medical Research Council Policy on data, software and materials management and sharing, all data supporting this study will be openly available.
