# Supplementary material for: Direct m6A recognition by IMP1 underlays an alternative model of target selection for non-canonical methyl-readers
Source: Nucleic Acids Res. 2023 Jun 28;51(16):8774–86. doi: 10.1093/nar/gkad534 (PMC10484666; doi:10.1093/nar/gkad534)
Supplement: gkad534_Supplemental_File [file gkad534_supplemental_file.pdf]

SUPPLEMENTARY DATA

Direct m6A recognition by IMP1 underlays an alternative model of target selection for non-canonical methyl-readers.

Giuseppe Nicastro[1*], Giancarlo Abis[2*], Pierre Klein[2], Sofia Esteban-Serna[2], Christopher Gallagher[2], Belen Chaves-Arquero[2], Yuyang Cai[2], Angelo Miguel Figueiredo[2], Stephen R. Martin[3], Rickie Patani[4], Ian A. Taylor[1#], Andres Ramos[2#].

[1] Macromolecular Structure Laboratory, The Francis Crick Institute, 1 Midland Road, London NW1 1AT, UK.
[2] Division of Biosciences, Institute of Structural and Molecular Biology, University College London, London, UK.
[3] Structural Biology Technology Platform, The Francis Crick Institute, 1 Midland Rd, London NW1 1AT, United Kingdom
[4] Human Stem Cells and Neurodegeneration Laboratory, The Francis Crick Institute, 1 Midland Road, London NW1 1AT, UK.
* These authors contributed equally
[#] Correspondence to a.ramos@ucl.ac.uk and ian.taylor@crick.ac.uk

	Protein	RNA
**NMR distance and dihedral constraints**		
Distance restraints		
Total NOE	2483	161
Unambiguous	2294	
Ambiguous	189	
Hydrogen bonds	58	
Protein–nucleic acid intermolecular		67
Total dihedral angle restraints		
Protein		
ϕ	103	
ψ	102	
Nucleic acid		
Sugar pucker		5
Backbone		17
**Structure statistics**		
Violations (mean and s.d.)		
Distance constraints (Å) (>0.3Å)	2	
Dihedral angle constraints (º)	0	
Max. dihedral angle violation (º)	0	
Max. distance constraint violation (Å)	0.357 ± 0.02	
Deviations from idealized geometry		
Bond lengths (Å)	0.019 ± 0.001	
Bond angles (º)	1.8 ± 0.02	
Impropers (º)	0.210 ± 0.02	
Average pairwise r.m.s. deviation** (Å)		
Protein		
Heavy	1.0 ± 0.1	
Backbone	0.6 ± 0.08	
RNA		
All RNA heavy		0.82 ± 0.18
Complex		
All complex heavy (C, N, O, P)	1.33 ± 0.2	

**Structural statistics were computed for ensemble of 20 deposited structures using PSVS 1.5. Ordered residues ([S(phi) + S(psi) > 1.8]): residues: 403–445, 450–479, 485–504, 508–524, 528–540, 543-565.

Supplementary Table 1. NMR and refinement statistics for the IMP1 KH(3)4-UCGG(m6A)CU RNA complex.

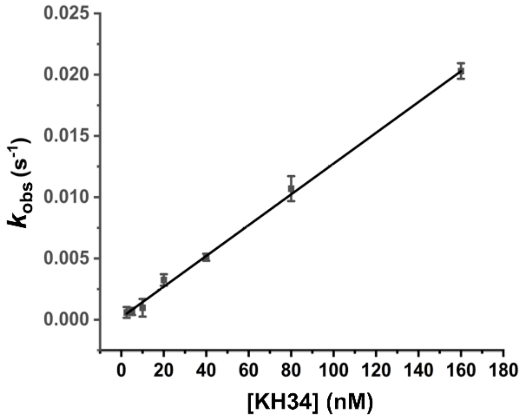

Supplementary Figure 1. BLI experiments. Representative plot from kinetics analyses. k_{obs} values were obtained in a titration of immobilised methylated ACTB mRNA Zipcode with increasing concentrations of IMP1 KH34 di-domain and plotted against the protein concentration. The plot was used to derive the k_{on} to calculate the K_D values.

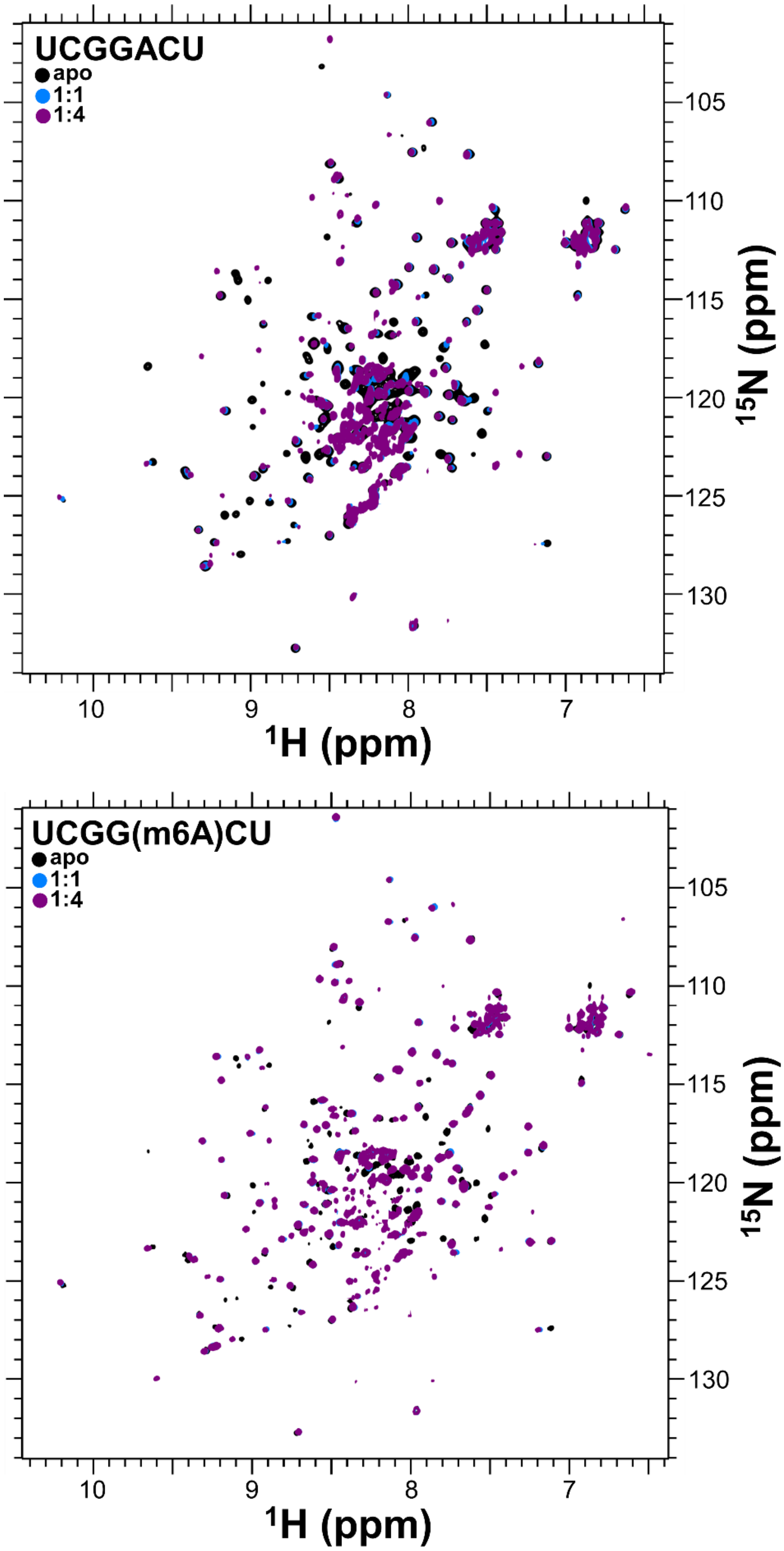

Supplementary Figure 2. Titration of KH(3)4 protein with UCGGACU and UCGG(m6A)CU RNAs. Overlay of ^{1}H-^{15}N HSQC spectra of KH(3)4 free – black – and bound to UCGGACU (top) and UCGG(m6A)CU (bottom) RNAs at molar ratios of 1:1 – light blue – and 1:4 – purple. The plots display the full amide region of the spectra.

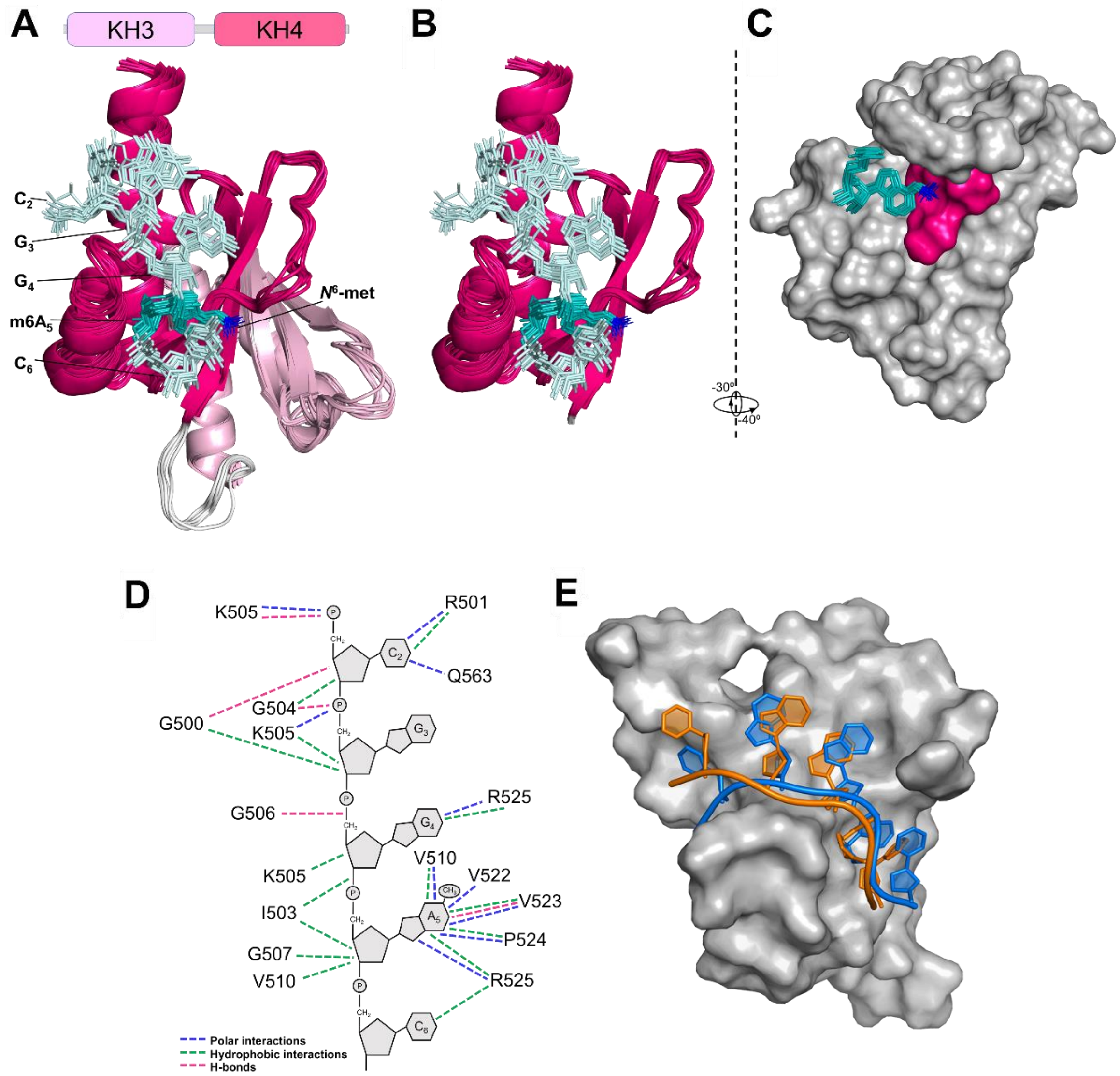

Supplementary Figure 3. Structural analysis of protein-RNA interactions. (**A**) Superimposition of the 10 lowest energy structures of the ensemble calculated for the KH(3)4-UCGG(m6A)CU complex (PDB code 8COO). The protein is displayed in cartoon representation, with the domains colour coded as in the schematic above. The physiological five-nucleotide core RNA sequence (CGGAC) is displayed in stick representation and coloured in teal, with the m6A nucleotide in a darker tone. (**B**), As in (A), but only KH4 is represented, for clarity. (**C**) A m6A-focused representation of the same structural bundle highlights how the position of the modified nucleotide is well defined in the structure. The protein surface is shown in grey, with the three amino acids of the hydrophobic cradle coloured in dark magenta. The ensemble of structures for the m6A nucleotide are shown as sticks, coloured as in **A**. (**D**) KH(3)4 – UCGG(m6A)CU contacts observed in the protein-RNA complex. Hydrogen bonds are represented in magenta, hydrophobic interactions in green and electrostatic interactions in blue. The contacts reported are observed in the majority of structures in the converged ensemble (PDB code 8COO). (**E**) Comparison of the conformations of the bound UCGG(m6A)CU (blue) and UCGGACU (orange) RNA. The same nucleotides shown in **A** are displayed and the two RNAs are represented as stick while the protein surface is shown in grey (PDB codes 8COO, 2N8M).

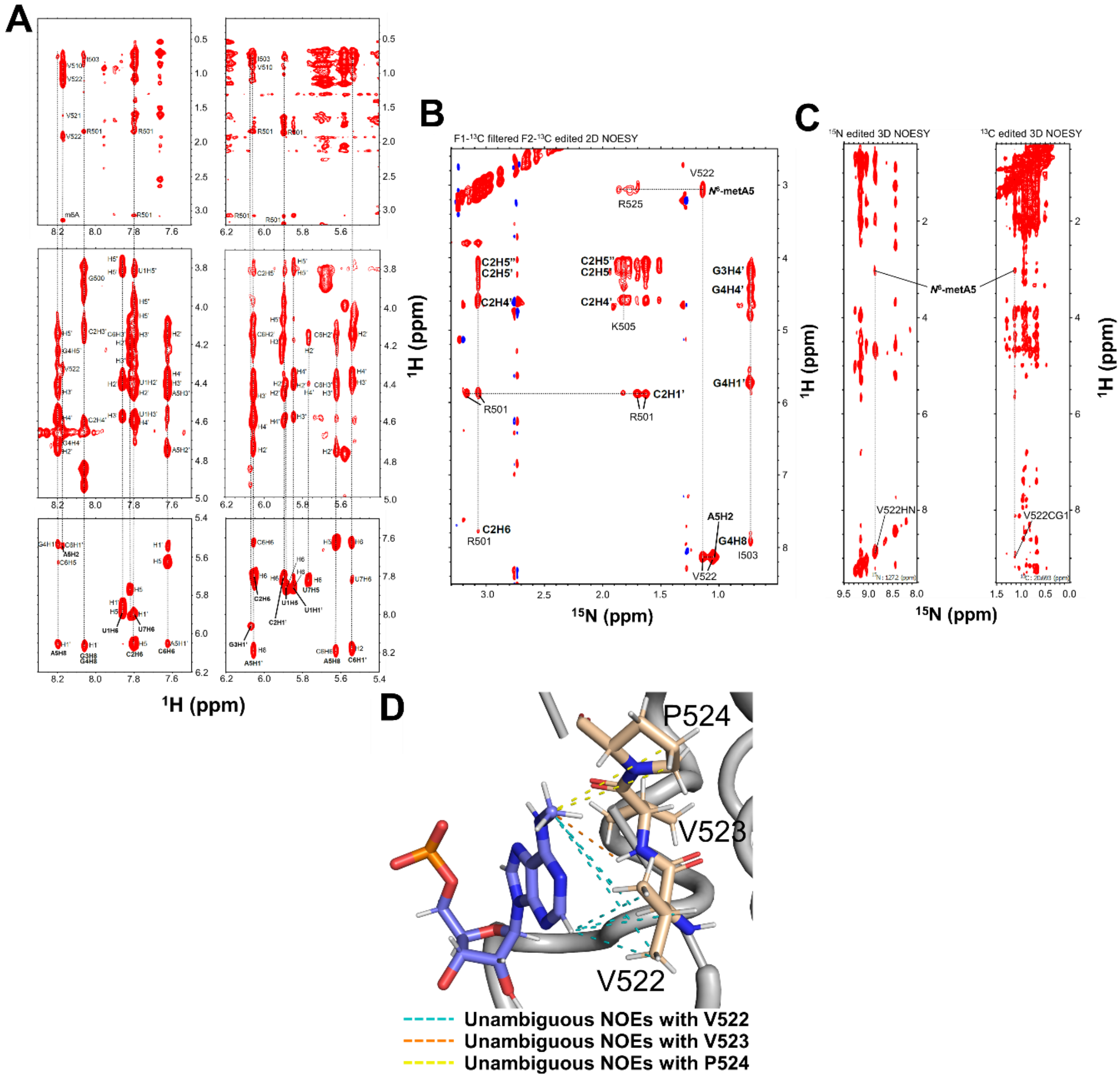

Supplementary Figure 4. NMR observables defining the protein-RNA interaction. (**A**) Homonuclear ^{1}H-^{1}H NMR spectrum recorded on the KH(3)4-UCGG(m6A)CU complex. A number of intermolecular NOE cross-peaks are highlighted to provide an assessment of data quality. Several intra-molecular NOEs are also labelled for comparison. (**B**) 2D-(^{1}H-^{1}H) F1-^{13}C filtered, F2-^{13}C edited NOESYs spectra recorded on the KH(3)4-UCGG(m6A)CU complex, displaying a number of representative NOEs cross-peaks, as well as various intra-molecular NOEs for comparison. (**C**) 2D ^{1}H-^{1}H planes from a 3D ^{15}N-edited NOESY (left) and a 3D ^{13}C-edited NOESY spectra are displayed to showcase data quality and highlight NOE cross-peaks to the methyl group of the m6A nucleotide. (**D**) Unambiguous NOEs signals between protein and m6A observed in the NMR experiments and used for the structure calculation.

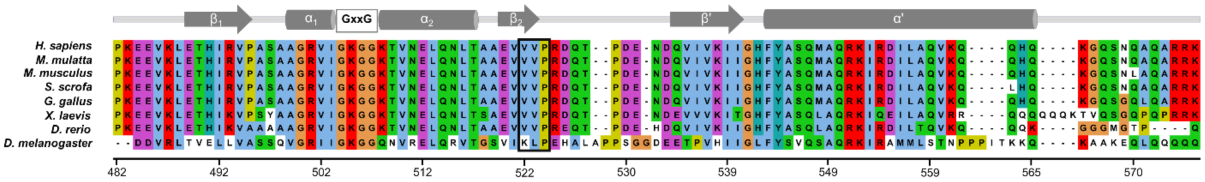

Supplementary Figure 5. IMP1-KH4 alignments. CLUSTAL X alignment of the KH4 sequences from *H. sapiens*, *M. mulatta*, *M. musculus*, *S. scrofa*, *G. gallus*, *X. laevis*, *D. rerio* and *D. melanogaster*. A cartoon of the secondary structure elements is shown above the sequences. The *G. gallus* KH34 di-domain, which is 98% identical to human protein, was used in this study. The *H. sapiens* numbering is reported at the bottom of the alignment and used throughout the paper, unless otherwise specified.

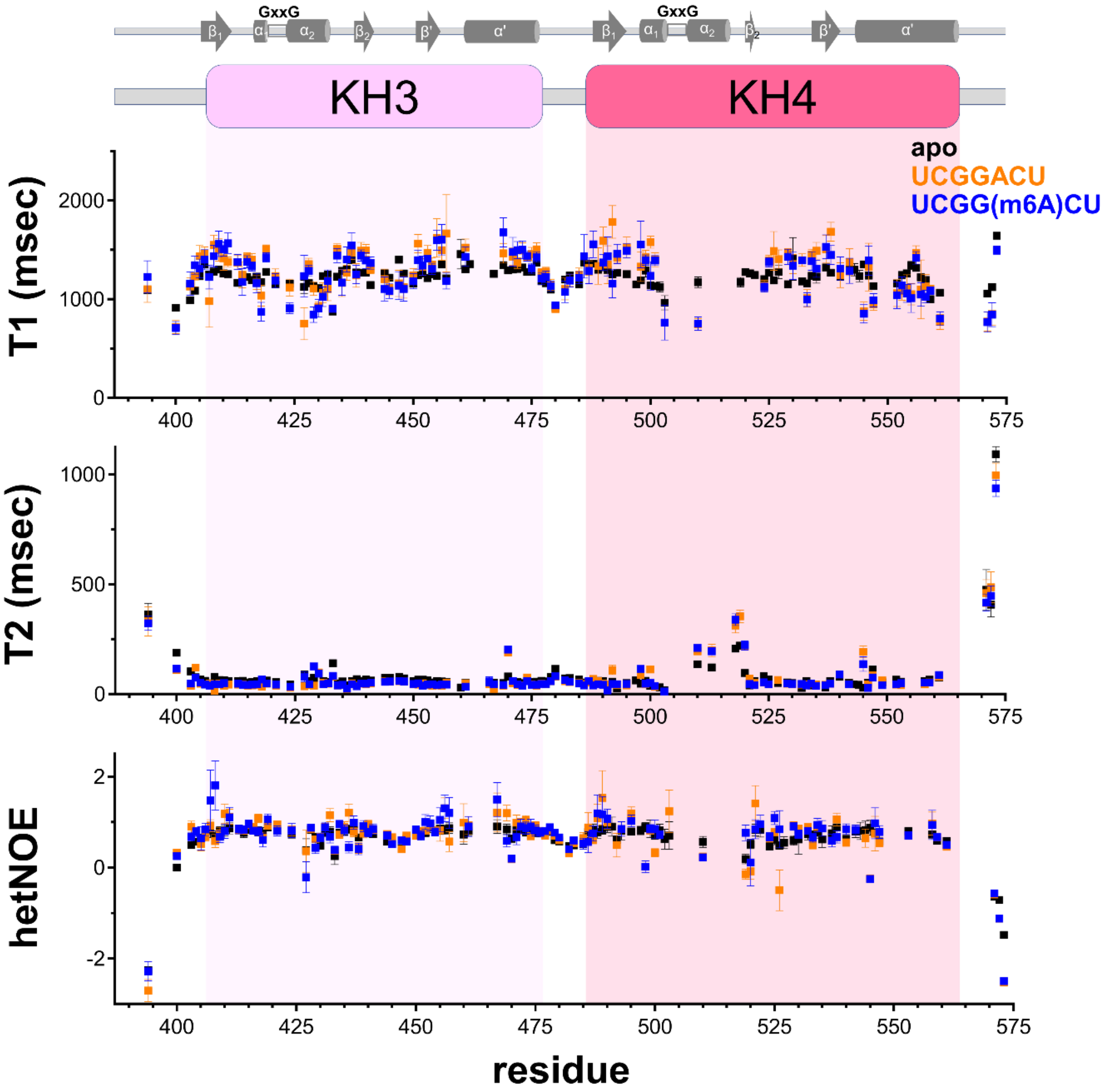

Supplementary Figure 6. Backbone amide dynamics of KH(3)4 free and bound to methylated or un-methylated RNA. ^{15}N T1 (top), ^{15}N T2 (middle) and $^{15}N\{^{1}H\}$-heteronuclear NOE (bottom) values for the protein backbone amides are plotted against residue number. KH3 and KH4 are highlighted using light pink and salmon shadowing. Values for apo protein and complexes with UCGGACU and UCGG(m6A)CU are reported in black, orange, and blue respectively. The small increase in overall T1 and T2 values is consistent with the small increase in molecular weight upon RNA binding. Secondary structure elements are displayed on the schematic above. Residues are labelled using *G. gallus* numbering.

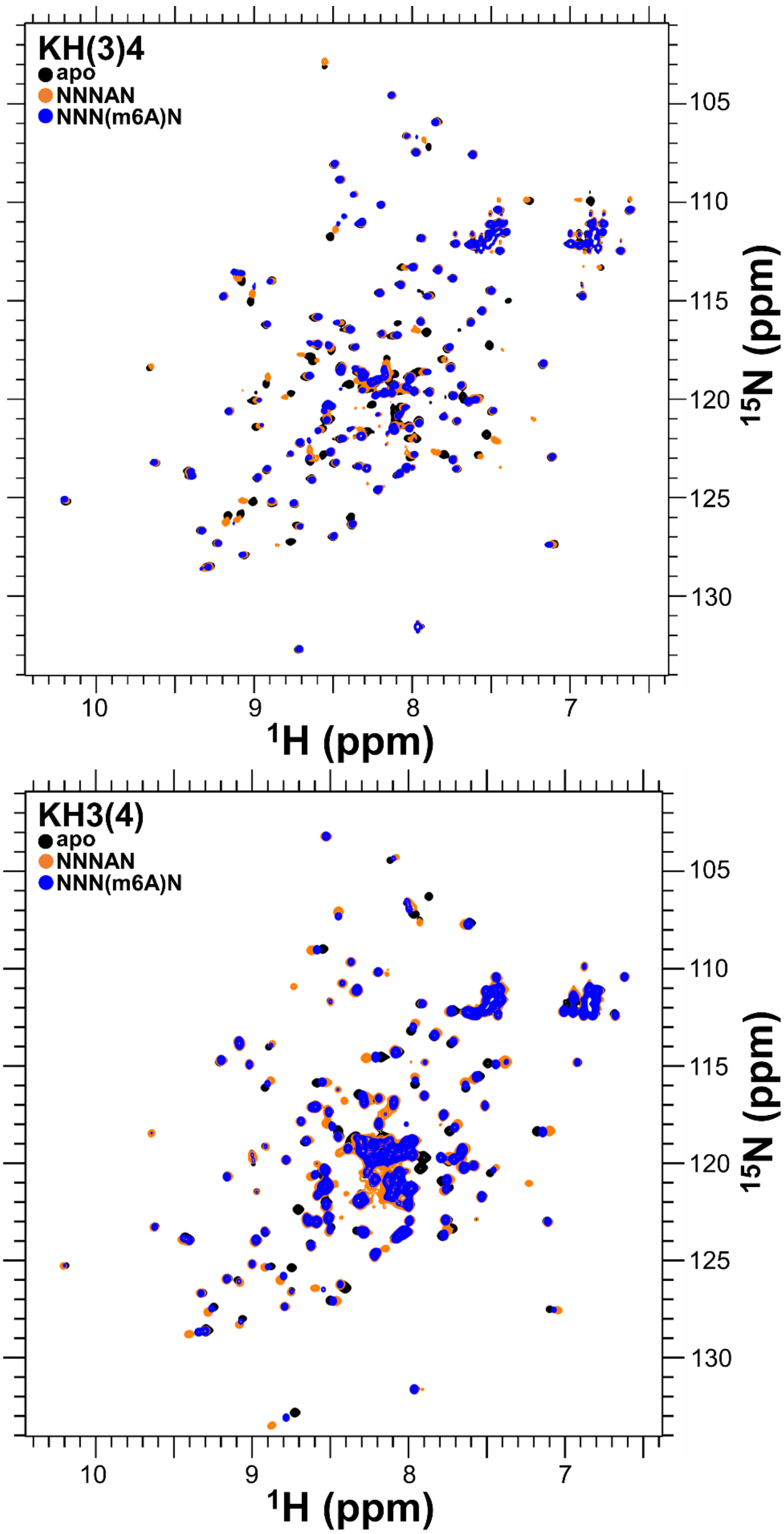

Supplementary Figure 7. Titrations of KH(3)4 and KH3(4) with quasi-randomised RNA pools. Overlay of ^{1}H-^{15}N HSQC spectra of free KH(3)4 (top) and KH3(4) (bottom) and bound (molar ratios 1:2) to quasi-randomised RNA pools. The apo protein spectrum is shown in black, while the complexes with NNNAN and NNN(m6A)N are in orange and blue respectively.

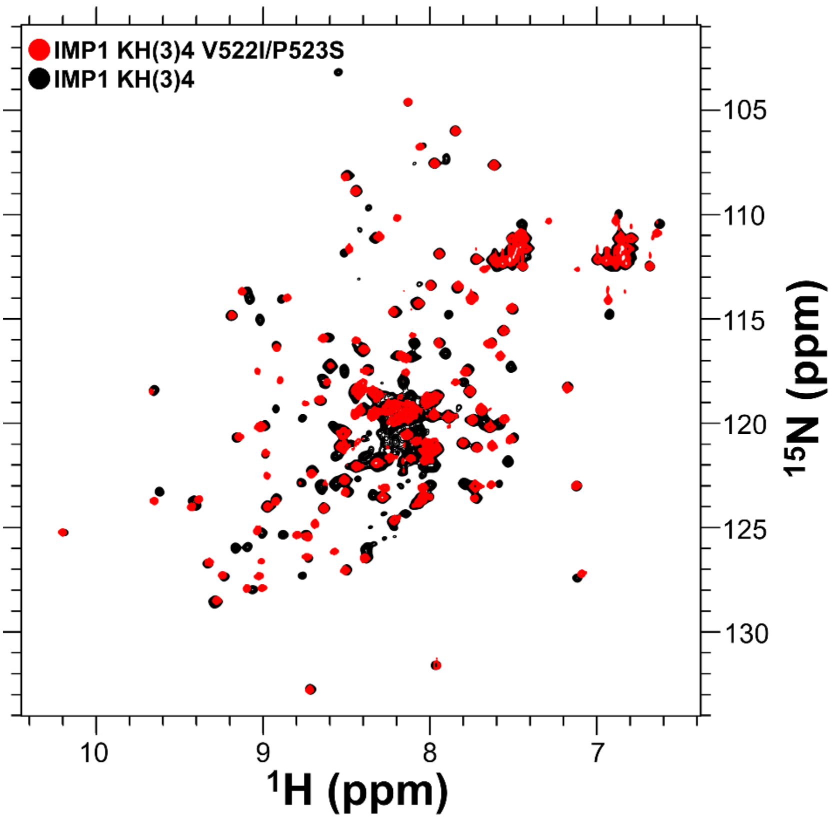

Supplementary Figure 8. Overlay of ^{1}H-^{15}N HSQC NMR spectra of KH(3)4 and KH(3)4 V523I/P524S double mutant. ^{1}H-^{15}N HSQC spectra of the KH(3)4 protein and its V523I/P524S mutant, are shown in black and red respectively. The limited differences indicate that the global structure of the di-domain does not change significantly in the double mutant.

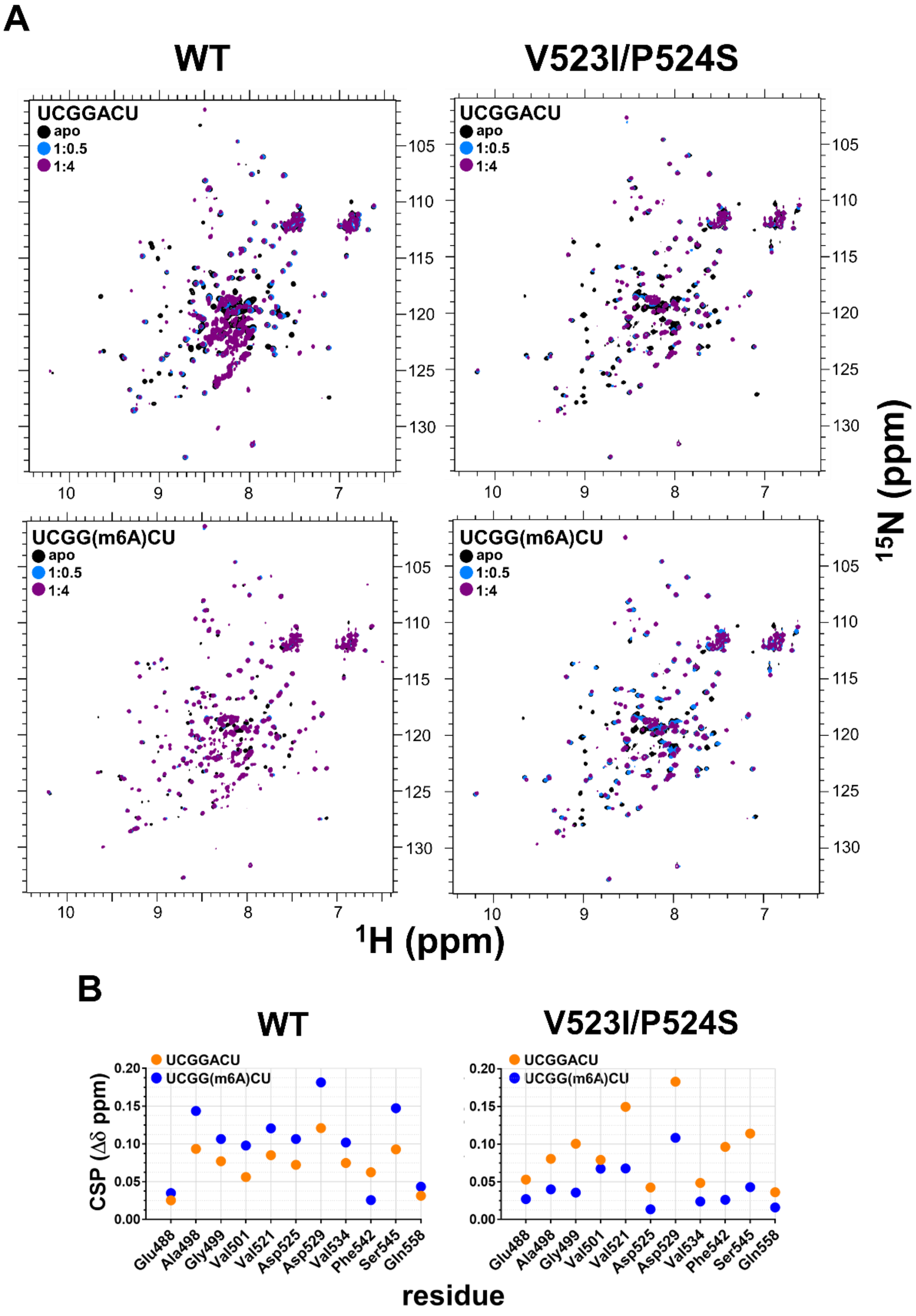

Supplementary Figure 9. (A) Binding studies of KH(3)4 and V523I/P524S double mutant. Overlay of ^{1}H-^{15}N HSQC spectra of free KH(3)4 (left) and KH(3)4 V523I/P524S double mutant (black) and bound (molar ratios 1:0.5 – light blue – and 1:4 – purple) to UCGGACU and UCGG(m6A)CU. (**B**) Quantification of chemical shift perturbations (CSP) of WT and V523I/P524S double mutant. The plots report the combined CSP observed at protein-to-RNA molar ratio of 1:0.5 for both KH(3)4 (left) and KH(3)4 V523I/P524S double mutant (right). The two constructs show opposite trends for the methylated and un-methylated RNA, validating the role of the ‘hydrophobic cradle’ in the coordination of the *N*^{6}-methyl group of m6A.

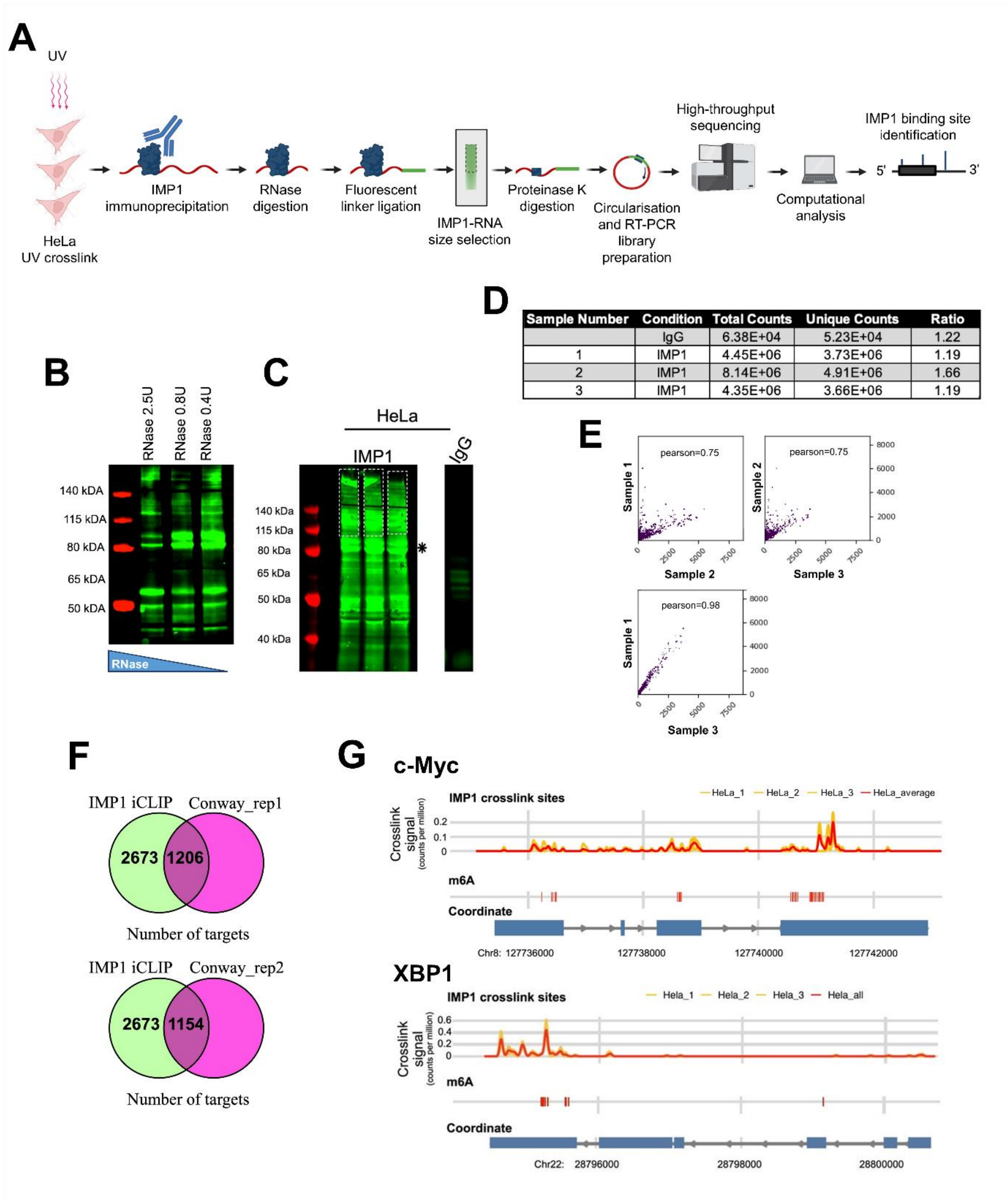

Supplementary Figure 10. Extended iCLIP data analysis. (**A**) Schematic of IMP1 iCLIP strategy in HeLa cells. Three technical replicates were UV-C crosslinked, lysed, and total RNA was fragmented with RNase I. IMP1-RNA complexes were immunoprecipitated and bound RNA was ligated to an oligo containing a fluorescent InfraRed dye. IMP1-RNA complexes were separated by SDS-PAGE and transferred to a nitrocellulose membrane. RNA was visualised by imaging IR dye fluorescence and the region of interest was extracted from the membrane. After proteinase K digestion RNA was reverse transcribed, circularised, and PCR amplified to generate sequencing libraries. Bioinformatics methods were applied to assess the exact binding location of IMP1 onto RNAs. (**B**) Representative LI-Cor scanning visualisation of nitrocellulose membrane of RNA/IMP1 complexes in HeLa cells treated with increasing

concentrations of RNase I, per 1 mL of lysate at 1 mg/mL protein concentration. (**C**) Representative LI-Cor scanning visualisation of nitrocellulose membrane of RNA/IMP1 and RNA/IgG complexes in HeLa using the optimised amount of 0.8 U of RNase I (see (B)), for 1 mg of total cellular protein. The excised portion of the membrane used to generate iCLIP libraries is shown within the dashed boxes. The asterisk represents the migration position of IMP1-RNA- L3IR adaptor complex. Both in **B** and **C**, visualisation is based on the infrared adaptor ligated to RNA. (**D**) Number of total reads per iCLIP sample. IgG is reported as a negative control. (**E**) Scatter plot representation of the correlation matrix for read genomic coverages for each sample. The numbers presented are the correlation coefficients for each comparison. (**F**) Venn diagram representing the overlap between genes bound by our IMP1 dataset and the Conway et al IMP1 dataset (refer to 56 in the main text) for each replicate. (**G**) Representative iCLIP and miCLIP tracks of the c-Myc and X-box binding protein 1 (XBP1) mRNAs. For the iCLIP tracks the signals for each HeLa replicate are shown in yellow and the merged signal from all three replicates in red. On the miCLIP tracks, a red line shows the presence of a detected m6A-site.

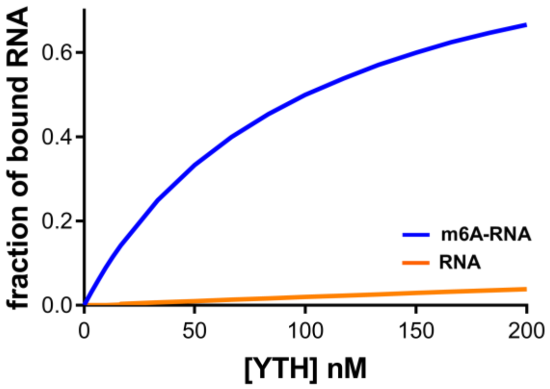

Supplementary Figure 11. Fraction of ACTB RNA bound to a YTH domain reader (as a model system), as a function of YTH concentration. Fraction of ACTB RNA Zipcode bound to YTH as a function of YTH concentration, at K_D values of 100 nM for the unmodified RNA (blue) and 5000 nM for the modified one (orange) (reference 7 in the main text). The total concentration of ACTB RNA Zipcode, used in these calculations is 0.4 nM, as for **Figure 5D**.
